# Human Organoids in Radiation Research: Modeling Tissue Injury, Regeneration and Therapeutic Response

**DOI:** 10.3390/ijms27219891

**Published:** 2026-11-04

**Authors:** Xiaolu Hao, Zhaohui Li, Shaojun Wang

**Affiliations:** 1 Senior Department of Ophthalmology, Chinese PLA General Hospital, Beijing 100039, China; oliviahxl@sina.com; 2 Medical School, Chinese PLA General Hospital, Beijing 100039, China

**Keywords:** organoids, radiation injury, tissue regeneration, radiosensitivity, radioprotection

## Abstract

Radiation therapy is a cornerstone in the treatment of abdominal, pelvic, thoracic, head and neck and central nervous system malignancies. However, irradiation inevitably damages adjacent normal tissues, leading to radiation-induced enteritis, lung injury, cardiac injury, salivary gland dysfunction and brain injury, which compromise both quality of life and treatment outcomes. Traditional two-dimensional cell cultures and animal models have significant limitations in mimicking human tissue radiation responses: the former lacks three-dimensional architecture and cell–cell interactions, while the latter exhibits species-specific differences. In recent years, three-dimensional organoid technology has rapidly emerged as a powerful platform in radiation biology and radiation oncology. In particular, organoids derived from patient tissues or induced pluripotent stem cells (hiPSCs) offer key advantages, including self-organization capacity and near-physiological tissue structure and function, which have greatly advanced in-depth research in the radiation field. This review comprehensively summarizes applications of human organoids in radiation injury and regeneration research across the brain, heart, lung, esophagus, salivary gland, intestine, muscle, thyroid, hematopoietic system, and tumors. Animal-derived and organotypic models are included only as comparative or supporting mechanistic evidence and are explicitly identified as such. We focus on advances in modeling radiation injury, elucidating regenerative mechanisms, screening radioprotective and radiosensitizing agents and predicting patient responses to chemoradiation. Current limitations and future directions are discussed collectively at the end.

## 1. Introduction

Ionizing radiation damages DNA directly and indirectly through reactive oxygen species, causing cell death, senescence, barrier disruption and chronic inflammation. Research on normal tissue radiation injury has long relied on animal models, but significant differences in radiosensitivity, immune responses, and regenerative capacity between mice and humans limit translational value. Organoids, as near-physiological three-dimensional culture systems, recapitulate organ-specific cellular composition, tissue architecture, and partial function, offering new opportunities for studying radiation injury and repair in vitro [1].

Organoid technology has developed along three main routes that differ in origin, genomic stability and application. Adult stem cell (ASC)-derived organoids are established from tissue-resident stem cells, retain the donor’s genome and are well-suited to normal-tissue toxicity studies. Induced pluripotent stem cell (iPSC)-derived organoids allow access to otherwise inaccessible tissues (e.g., brain, heart) and permit gene editing, but are typically less mature. Patient-derived tumor organoids (PDOs) retain the genomic alterations and histological features of the primary tumor, making them useful for both normal-tissue toxicity studies and for predicting individualized chemoradiation responses [2,3,4]. Importantly, these models differ in maturity, cellular composition and microenvironmental complexity, and findings obtained in one system should not be generalized to another without validation.

Beyond model choice, three mechanistic concepts are central to organoid-based radiation research and are developed throughout this review. Epigenetic reprogramming allows radiation-injured epithelia to re-enter a fetal-like regenerative program [5]. Stem-cell competition shapes which clones repopulate a tissue after injury and is sensitive to dose rate [6,7]. Bystander effects describe biological changes in non-irradiated cells receiving signals from irradiated neighbors, and have been documented even at low doses of high-LET radiation [8]. Organoids uniquely permit these processes, lineage trajectories, niche crosstalk, clonal dynamics and non-targeted effects to be interrogated in human three-dimensional tissue rather than in monolayer cultures [9,10]. At the same time, organoids remain an incomplete model, and this review therefore appraises their strengths and limitations critically rather than celebratorily.

This review is narrative in scope. Relevant literature was identified through PubMed and Web of Science using combinations of the terms “organoid”, “radiation”, “radiotherapy”, “radiosensitivity”, “radioprotection” and organ-specific terms. Studies based on human organoids are the primary focus, whereas animal-derived organoids, organotypic slice cultures and two-dimensional systems are cited only where they provide mechanistic or comparative evidence and are identified accordingly. The review summarizes major applications of organoids in radiation research by organ system (summarized in Table 1), with limitations and improvement strategies discussed collectively at the end.

## 2. Brain Organoids

Brain organoids provide a human three-dimensional platform for studying radiation effects on neurogenesis, synaptic integrity and neuroinflammation. Shakhbazau et al. found that rapamycin or minocycline partially rescued neuroepithelium integrity, neurite outgrowth, ventricle formation, and extracellular acidification rate after irradiation [11]. Das et al. established human forebrain organoids and quantified γ-H2AX and 53BP1 foci repair kinetics in neural progenitors and mature neurons after 2 Gy irradiation, finding that DNA double-strand breaks were mostly resolved by 18 h, with more acute repair kinetics in progenitors [12]. Bojcevski et al. further demonstrated that DNA damage repair kinetics in hiPSC-derived brain organoids can serve as a surrogate for normal tissue radiosensitivity [13]. Oyefeso et al. exposed brain organoids to 0.5 or 2 Gy proton radiation and found time- and dose-dependent increases in DNA damage, with the downregulation of oligodendrocyte lineage, astrocyte lineage, mitochondrial function, and cell cycle genes at 48 h, and the upregulation of neuron lineage, oxidative stress, and DNA damage checkpoint genes [14]. Lundberg et al. showed that clonidine significantly reduced radiation-induced loss of neural progenitor cells, neurons, astrocytes, and oligodendrocyte lineage cells, and mitigated DNA damage and reactive gliosis [15]. Shimada and Matsumoto demonstrated that DMSO and ascorbic acid administered before or after irradiation alleviated DNA damage, cell death, and microcephaly phenotypes [16]. He et al. used mature human cortical organoids to study acute and delayed effects of single-dose and fractionated irradiation, revealing that radiation not only reduced viability but also reshaped lineage trajectories, with the depletion of neural stem/progenitor populations, loss of neuronal identity, enhanced gliogenesis, increased inflammatory cytokines, and disrupted cortical layering and synaptic integrity; NSPP and amisulpride attenuated these changes [17]. Jiang et al. compared photon, plateau proton, and spread-out Bragg peak (SOBP) proton irradiation and found that SOBP protons uniquely downregulated brain development and synaptic signaling genes and impaired neural network function [18]. Not all brain models are equivalent. Kay et. al. used a rat brain organotypic slice co-cultured with breast carcinoma cells, not a human brain organoid, to study FLASH effects, finding that HIGS-FLASH irradiation inhibited tumor growth while causing differential changes in cytokines and microglial morphology [19]. These data are therefore treated as comparative animal evidence, and their relevance to human brain organoids remains to be established.

Brain organoids represent one of the fastest-growing and most readout-rich directions in radiation research, yet their current prominence should be assessed critically rather than celebratorily. Their real significance lies not simply in adding a human three-dimensional model, but in challenging the long-standing dominance of animal models and two-dimensional cell cultures in radiobiology. By preserving human genetic background, cytoarchitecture and cell–cell interactions, brain organoids allow radiation effects to be interrogated at the level of neural lineage trajectories, synaptic integrity, neuroinflammation and network function-readouts that are difficult to capture adequately in conventional systems. Notable advances include: first, the establishment of DNA damage repair kinetics as a surrogate for normal brain tissue radiosensitivity, which offers a human platform for individualized radiosensitivity assessment, although its predictive validity still requires clinical validation; second, the demonstration that radiation not only causes cell death but also reshapes neural lineage trajectories, leading to the depletion of neural stem/progenitor populations, loss of neuronal identity, enhanced gliogenesis, reactive gliosis and neuroinflammation, thereby reframing radiation injury as a developmental and cell-fate disorder rather than a simple cytotoxicity problem; third, comparisons across radiation qualities, dose regimens and FLASH irradiation, which reveal distinct biological effects such as SOBP proton-specific downregulation of brain development and synaptic signaling genes and differential cytokine/microglial responses under FLASH conditions, though whether these differences translate into clinically meaningful sparing or toxicity remains unresolved; fourth, drug rescue experiments (clonidine, DMSO, ascorbic acid, NSPP, amisulpride, rapamycin, minocycline) that provide candidate radioprotective agents and suggest that neuroprotection may require targeting DNA damage, oxidative stress, inflammation and lineage dysregulation simultaneously, but most of these findings remain preclinical, dose- and timing-dependent, and lacking validation in vivo.

These advances are constrained by substantial limitations. Brain organoids remain immature relative to the postnatal human brain, exhibit considerable batch-to-batch and line-to-line variability, lack a functional vascular system, blood–brain barrier and resident immune microenvironment, and often display regional heterogeneity that complicates cross-study comparison. Many radiation studies still rely on limited donor lines, single omics layers and short follow-up windows, raising questions about reproducibility and about whether the observed repair kinetics, lineage shifts or inflammatory responses faithfully mirror the adult human brain. The absence of standardized irradiation protocols, endpoint definitions and organoid maturity criteria further hampers quantitative synthesis, and the frequent use of supraphysiological doses or acute single exposures may not reflect clinically relevant fractionation or chronic exposure. Brain organoids should therefore be viewed as a powerful but incomplete complement to animal models and clinical data rather than a replacement. Future work should prioritize vascularized and immune-competent organoids, patient-derived and genetically diverse cohorts, standardized and clinically relevant irradiation schemes, multi-omics together with functional readouts such as electrophysiology and neural network activity, and systematic cross-validation with in vivo and clinical endpoints.

## 3. Heart and Lung Organoids

The lung and heart reside in the thoracic cavity and are the normal organs most frequently irradiated during thoracic radiotherapy. Radiation-induced lung injury (RILI), radiation-induced pulmonary fibrosis (RIPF), and radiation-induced heart disease (RIHD) are major dose-limiting complications of thoracic radiotherapy. Both are studied using three-dimensional models derived from iPSCs or tissue stem cells to assess structural disruption, functional decline, and pharmacological intervention.

For lung organoids, Budeus et al. generated matrix-free, free-floating human lung organoids from iPSCs that morphologically and cellularly resemble human lung target tissue, revealing differential radiosensitivity among lung cell types and senescence-associated gene sets after irradiation [1]. Kuipers et al. used air–liquid interface-differentiated primary human bronchial epithelial cells and found that organoid formation capacity was significantly reduced after irradiation and persisted for at least 7 days, despite no apparent cytotoxicity, inflammation, or EMT marker elevation, suggesting that organoid formation capacity is a sensitive readout for normal lung epithelial radiation injury [20]. Park et al. established an hESC-derived human lung organoid model containing AT1, AT2, goblet, basal, club, and ciliated cells. After repeated irradiation, organoids exhibited key RIPF features including impaired proliferation, epithelial barrier disruption, EMT, upregulation of profibrotic cytokines, and collagen deposition; single-cell RNA sequencing revealed reduced proliferative AT2 cells and altered epithelial subpopulations. The antifibrotic drug pirfenidone significantly reduced TGF-β, α-SMA, and COL1A2 expression after irradiation [21]. Xuan et al. combined transcriptomic and metabolomic analysis of lung organoids and found that 0.01 Gy low-dose radiation affected glycerophospholipid metabolism pathways [22]. For cardiac organoids, Cao et al. established an X-ray-induced cardiac organoid injury model and identified 20 Gy as the optimal dose for this model. This dose is specific to the experimental system and does not represent clinical cardiac radiation exposure. After irradiation, cardiac organoids showed increased cell death, decreased energy metabolism, altered calcium transients, disrupted mitochondrial structure, and reduced membrane potential; small extracellular vesicles (sEVs) from umbilical cord mesenchymal stem cells inhibited these effects, possibly through regulating oxidative phosphorylation and p53 signaling [23].

Lung and cardiac organoids currently provide short-term readouts dominated by a single cell type. They lack pulmonary immune populations, microvascular perfusion and cardiac fibroblast–endothelial coupling, and most studies use single high-dose exposures rather than clinically relevant fractionation. Whether molecular endpoints such as collagen deposition or calcium transients quantitatively predict organ-level functional injury in patients remains to be established. Within these constraints, lung organoids have advanced the field by avoiding animal-derived matrices and species differences, enabling the direct quantification of normal lung epithelial radiosensitivity in human cells by recapitulating key molecular and structural features of fibrosis under repeated irradiation for antifibrotic drug screening, and by establishing organoid formation capacity as a more sensitive readout than traditional toxicity indicators. Cardiac organoids have provided a three-dimensional model of radiation-induced cardiac injury with multi-level functional readouts, including viability, energy metabolism, calcium transients and mitochondrial ultrastructure, and have shown that stem cell-derived EVs exert protective effects through metabolic and p53 pathways.

## 4. Esophagus and Salivary Gland Organoids

The esophagus and salivary glands are located within head and neck or upper gastrointestinal radiation fields and are common target organs of radiation injury. Radiation exposure of the esophagus is associated with acute esophagitis and chronic stricture, and in the long-term, with an increased risk of esophageal carcinogenesis; radiation-associated carcinogenesis should be distinguished from the far more common acute and chronic non-malignant complications of esophageal irradiation. Radiation-induced xerostomia is one of the most common long-term complications of head and neck radiotherapy. Both are studied using three-dimensional models derived from tissue stem cells or primary tissues to investigate epithelial differentiation, tissue architectural disruption, and transplantation repair potential.

For esophageal organoids, Carswell et al. used murine three-dimensional esophageal organoids to study radiation quality-and dose-dependent persistent changes [8]; these data are treated as comparative animal evidence. High-LET iron ion irradiation caused more intense and persistent alterations in squamous differentiation and 53BP1 foci than cesium irradiation, although most phosphoproteins in the MAPK/SAPK stress pathway showed similar changes; low-dose 0.1 Gy high-LET irradiation also caused nearly identical morphological changes despite only approximately 36% of cells predicted to be hit, suggesting bystander effects. Human esophageal organoid data remain limited, and the murine findings should not be assumed to translate directly. For salivary gland organoids, Jeon et al. isolated stem cells from human salivary gland tissue and cultured human salivary gland organoids in Matrigel and collagen. Collagen-cultured hSGOs maintained self-renewal capacity and more closely replicated salivary gland tissue characteristics after differentiation; xenograft transplantation resulted in detectable human-specific nucleoli and E-cadherin expression, and MUC5B expression suggested potential improvement in salivary composition. Allogeneic transplantation of mouse salivary gland organoids increased salivation [24], providing preclinical proof-of-principle for cell therapy.

Esophageal organoid work is still dominated by murine systems, and human esophageal organoid radiation data are scarce. Salivary gland organoid transplantation data derive largely from animal models, and long-term engraftment, functional durability and safety in patients remain unproven. Against this background, esophageal organoids have linked radiation quality (high-LET versus low-LET) to alterations in esophageal epithelial differentiation programs and have provided in vitro evidence of bystander effects, showing that even low-dose high-LET irradiation can cause persistent morphological changes in non-directly hit cells through bystander signaling. Salivary gland organoids have moved beyond Matrigel to collagen-based culture, addressing undefined composition and immunogenicity in clinical translation, and have shown that allogeneic transplantation can restore salivation, providing preclinical evidence for the cell therapy of radiation-induced xerostomia.

## 5. Intestinal Organoids

The intestinal epithelium renews rapidly and is highly radiosensitive. Abdominal or pelvic irradiation can cause intestinal stem cell (ISC) depletion, crypt destruction, and mucosal barrier loss, leading to radiation-induced gastrointestinal syndrome. Intestinal organoids retain crypt-villus architecture and Lgr5^+^ ISCs, making them a core model for studying radiation-induced intestinal injury and regeneration [26].

For radiation injury modeling, multiple studies have irradiated intestinal organoids with γ-rays or X-rays and observed viability loss and regrowth similar to in vivo responses. Montenegro-Miranda et al. established an in vitro damage–repair model by applying γ-radiation to small intestinal organoids and identified HNF4α as a critical upstream regulator of intestinal regeneration; Hnf4α knockout mice showed impaired regeneration after whole-body irradiation, validating the consistency of the organoid model with in vivo findings [25]. For stem cell protection and regeneration promotion, BCN057 rescued Lgr5^+^ ISCs after abdominal irradiation, promoted epithelial regeneration, and reduced mortality while providing no radioprotection to colon tumor tissue, suggesting that it may improve the therapeutic ratio of abdominal radiotherapy [26]. In mice, NOD2 deficiency was associated with significantly decreased crypt survival and organoid formation capacity after irradiation. Me6TREN promoted ISC proliferation and crypt regeneration by activating β-catenin signaling [27]. SIRT1 inhibition stabilized p53 through increased acetylation, improving the survival of mouse and human intestinal organoids after irradiation and increasing the survival of lethally irradiated mice from 0% to 88.89% [28].

For microenvironment and immune regulation, MSC-CM promoted ISC reproliferation and the restoration of damaged crypt-villus structures after irradiation, associated with Wnt/Notch signaling upregulation [29]. ILC-derived IL-22 directly targeted Lgr5^+^ ISCs in a STAT3-dependent manner, promoting mouse and human intestinal organoid growth and in vivo regeneration after transplantation [30]. Macrophages recruited to the ISC compartment after radiation injury promoted fetal-like reprogramming and epithelial proliferation through Nrg1 and Spp1; macrophage ablation compromised regeneration [31]. VIP promoted secretory differentiation through p38 MAPK and mitigated radiation-induced intestinal injury [32]. WNT4 negatively regulated canonical Wnt/β-catenin signaling in a ROR2-dependent manner, promoting symmetric crypt fission and improving regenerating crypt regularity [33]. Microbial metabolite I3A promoted epithelial proliferation and increased probiotic abundance through the AhR/IL-10/Wnt pathway [34]. NR inhibited GSDME-mediated pyroptosis in a SIRT1-dependent manner, restoring intestinal epithelial homeostasis [35]. For radiation dose, dose rate, and epigenetics, OFP reflects stemness: after high-dose-rate irradiation, crypt stem cell percentage decreased but OFP increased at total doses >1 Gy; 0.1 Gy high-dose-rate or 1 Gy low-dose-rate irradiation did not alter stemness [6]. Low-dose-rate irradiation also induced stem cell competition [7]. Qu et al. established hyper-organoids that model injury-associated hyperplastic crypt regeneration through VPA and EPZ6438-mediated epigenetic reprogramming and YAP signaling [5]. Additionally, low-dose nanoplastics aggravated radiation-induced intestinal injury through the TGF-β1/p-Smad3 pathway [46]; human intestinal organoids showed distinct molecular responses to proton versus photon radiation, with γ-irradiation inducing fetal-associated transcription factors and serotonin biosynthesis as regenerative nodes [36]; IL-22 levels correlated with intestinal radiosensitivity, with high-IL-22 mice exhibiting more severe intestinal injury [47]. IL-22 has been reported to both promote intestinal regeneration [30] and to correlate with more severe radiation injury [47]. These observations are not necessarily contradictory, because the net effect of IL-22 depends on context, dose, timing and target cell. IL-22 acts on Lgr5^+^ ISCs via STAT3 to promote epithelial repair after moderate injury, whereas sustained or excessive IL-22 signaling, or IL-22 acting on non-epithelial compartments, may amplify inflammatory and tissue-remodeling responses and thus correlate with greater injury severity. The direction of effect therefore cannot be inferred from expression levels alone and requires functional testing in controlled organoid systems.

Intestinal organoids represent the most mature and mechanistically dissected direction in radiation research, largely because the intestinal epithelium offers a well-defined stem-cell hierarchy, a rapid and quantifiable regenerative readout, and a close correspondence between organoid phenotypes and in vivo radiation responses. Notable advances include: first, incorporating immune cells, neurons, mesenchymal cells, and microbial metabolites of the ISC niche into the radiation regeneration research framework, demonstrating that regeneration is not an epithelial cell-autonomous process but is co-regulated by IL-22, macrophages, VIP, WNT4, and other microenvironmental signals; second, establishing that radiation injury and regeneration are shaped by reciprocal crosstalk between Lgr5^+^ ISCs and their niche, with MSC-CM, ILC-derived IL-22, macrophage-derived Nrg1/Spp1, WNT4/ROR2 signaling, and microbial metabolite I3A acting through the Wnt/β-catenin, STAT3, p38 MAPK and AhR/IL-10 pathways to promote stem cell reproliferation, crypt fission and epithelial restoration; third, introducing epigenetic reprogramming and stem cell competition into radiation response research through hyper-organoids and low-dose-rate organoid experiments, revealing that stemness, regenerative capacity and crypt dynamics are not fixed but can be remodeled by VPA/EPZ6438-mediated epigenetic changes, YAP signaling, dose rate and stem cell competition; fourth, linking radiation responses to clinically relevant modifiers such as SIRT1/p53 acetylation, GSDME-mediated pyroptosis, NOD2 deficiency, TGF-β1/p-Smad3 signaling and IL-22 levels, thereby identifying candidate targets for radioprotection and therapeutic ratio improvement, exemplified by BCN057’s selective protection of normal ISCs without shielding colon tumor tissue.

Nevertheless, critical limitations remain. Most mechanistic findings still derive from mouse-derived organoids or a limited number of human cell lines, and the absence of a full immune, neural, vascular and microbial microenvironment means that niche interactions are only partially reconstituted. Dose, dose-rate and fractionation schemes vary widely across studies, and endpoints such as organoid formation efficiency, crypt budding and viability are not fully standardized, complicating cross-study comparison and clinical extrapolation. Whether drug candidates such as BCN057, Me6TREN, SIRT1 inhibitors or I3A can achieve durable radioprotection without compromising tumor control in patients also remains to be established. Future work should prioritize patient-derived and genetically diverse intestinal organoids, co-culture or microfluidic systems incorporating immune and neural components, standardized and clinically relevant irradiation protocols, multi-omics and functional readouts, and systematic validation against in vivo and clinical endpoints.

## 6. Muscle Organoids

Skeletal muscle is relatively radioresistant, but high-dose irradiation can still cause muscle atrophy and impaired repair. Jiang et al. established hPSC-derived three-dimensional human skeletal muscle organoids (hSMOs) that recapitulate embryonic myogenesis and maturation. After 2 Gy irradiation, hSMOs exhibited deficits in expansion, differentiation, and repair response at early (30 min) and intermediate (18 h) time points, with elevated γ-H2AX and 53BP1 foci in PAX7^+^ cells that decreased by 72 h; RNA sequencing further revealed DNA damage response signatures [37].

This evidence comes from a single study based on one hPSC line. Myofiber innervation, immune infiltration and long-term functional (contractile) recovery were not assessed, and the 2 Gy single exposure does not represent clinical fractionation. The model nevertheless fills a gap in human three-dimensional systems for skeletal muscle radiation response. Previous studies relied on animal models or two-dimensional myotube cultures, which cannot simultaneously preserve myofiber structure, satellite cells and differentiation gradients. hSMOs have shown that PAX7^+^ cells are particularly radiosensitive and have provided DNA damage kinetics at different time points after irradiation, offering a new tool for studying muscle satellite cell radiosensitivity and protective strategies.

## 7. Thyroid Organoids

The thyroid gland is located within head and neck radiation fields and is susceptible to radiation injury. Green et al., as early as 2002, cultured Fischer rat thyroid cells in a low-shear stress bioreactor to form thyroid tissue organoids containing follicular structures; after 3 Gy γ-irradiation, tissue architectural disruption, actin disorganization, and loss of follicular content were observed, with increased active TGF-β release [38]. This is an animal-derived model and is presented as early proof-of-principle. Venegas et al. used CRISPR-Cas9 to introduce DTC-associated or non-associated germline PTEN mutations into healthy donor hiPSCs and induced functional thyroid organoids [39]. After irradiation, p53 network upregulation was stronger in PTEN WT/G132D organoids than in PTEN WT/M134R, possibly mediated by AKT-dependent MDM2 inactivation and PTEN-p53 interaction, explaining why PTEN M134R is a DTC-susceptible variant. Maturi et al. irradiated tissue-derived thyroid organoids and found that interferon-β signaling is a key mediator of radiation-induced thyroid inflammation, with intrinsic apoptosis as the predominant death mode [40]; IFN-β exhibited protective effects against apoptosis but reduced thyroid stem/progenitor cell potential.

Human thyroid organoid radiation studies remain few, derive from limited donor lines, and have not been validated against clinical thyroid radiosensitivity or carcinogenesis risk. The long-term follow-up required to link genotype-specific DNA damage responses to actual cancer incidence is not feasible in current organoid systems. Within these limits, thyroid organoids have connected radiation carcinogenesis susceptibility to genotype-specific DNA damage responses. Rather than studying thyroid cancer after tumor formation, Venegas et al. introduced germline PTEN variants into normal thyroid organoids and showed that different PTEN variants produce different p53 network activation intensities after irradiation, providing a mechanistic explanation for why only some patients with PTEN hamartoma tumor syndrome develop DTC. This work marks a transition of organoids from descriptive radiation injury models to genotype-driven radiation carcinogenesis risk models.

## 8. Hematopoietic Organoids

Acute hematopoietic radiation injury is a major complication of nuclear accidents and whole-body irradiation. Chen et al. obtained CD34^+^ hematopoietic stem/progenitor cells (HSPCs) from hESCs, mixed them with niche-related cells in GelMA hydrogel, and constructed three-dimensional hematopoietic organoids. GelMA maintained a HSPC undifferentiated state by reducing intracellular ROS; hematopoietic organoids exhibited radiation-induced hematopoietic cell injury including increased ROS, γ-H2AX-positive cell percentages, and apoptosis percentages; G-CSF supplementation significantly improved HSPC survival and myeloid regeneration after irradiation [41].

The model uses a single synthetic hydrogel formulation and a limited set of niche cells; it does not reconstitute bone marrow architecture, sinusoidal vasculature or the full hematopoietic niche, and has not yet been validated against patient marrow responses or long-term engraftment. Its value lies in the first assembly of hESC-derived HSPCs with niche cells in a synthetic hydrogel to form three-dimensional hematopoietic tissue for radiation injury and G-CSF efficacy assessment. By showing that GelMA maintains HSPC stemness through ROS reduction while supporting the recapitulation of radiation-induced hematopoietic injury phenotypes, it provides a scalable platform for drug screening and stem cell therapy in hematopoietic radiation injury.

## 9. Tumor Organoids

Applications of tumor organoids in radiation research mainly include two aspects: studying tumor cell radiation responses and microenvironmental interactions, and predicting patient chemoradiation responses. For tumor–microenvironment interactions, Feng et al. found that BM-MSCs irradiated with low-dose ultraviolet or X-rays inhibited colorectal cancer cell proliferation and induced apoptosis through the secretion of TNF-α and IFNγ [42]. Raguin et al. established vascularized complex cerebral organoids containing microvessels and microglia; co-culture with glioma stem cells recapitulated glioblastoma vascular co-option, microglial reprogramming into tumor-associated macrophages, and recurrence after radiotherapy [48]. For radiosensitivity mechanisms, Endo et al. used colorectal cancer organoids and found that sublethal irradiation drastically reduced proliferation and stemness markers while differentiation markers remained; after high-dose irradiation, regrowth foci appeared after a static phase, initiated by a small subset of cells with high Wnt activity [49]. Clifford et al. demonstrated that high acid ceramidase expression correlates with poor response to neoadjuvant treatment in locally advanced rectal cancer; AC knockdown or inhibition enhanced the radiosensitivity of colorectal cancer cells and patient-derived organoids, with mechanisms involving PARP-1 cleavage and p53-dependent apoptosis [50]. Yang et al. found that IDH1-mutant glioma organoids were more sensitive to radiotherapy and vorasidenib [51]. For patient response prediction, Yao et al. established a living organoid biobank from LARC patients enrolled in a phase III clinical trial, confirming that rectal cancer organoids closely recapitulate the pathophysiology and genetic changes of corresponding tumors; patient chemoradiation responses highly matched RCO responses with 84.43% accuracy, 78.01% sensitivity, and 91.97% specificity [3]. Xu et al. further found that cancer organoid responses to combined treatment predicted LARC patient clinical outcomes more faithfully than monotherapy, with 92.50% and 93.75% accuracy in discovery and validation cohorts, respectively [4]. Ganesh et al. established a rectal cancer organoid platform supporting individualized chemoradiation response research [52]. Pasch et al. used patient-derived tumor organoids combined with optical metabolic imaging to quantify treatment response and tumor heterogeneity at the single-cell level and prospectively predicted treatment response in a metastatic colorectal cancer patient [2]. Issing et al. established a head and neck tumor organoid biobank and found that TP53 loss alone was insufficient to promote radiation resistance, while HPV 16 E6/E7 mediated radiosensitivity through cell cycle arrest induction [43]. Reinhold et al. demonstrated that radiotherapy combined with photodynamic therapy exhibited tumor-selective cytotoxicity and synergistic effects in bladder cancer organoids and promoted T cell migration [44]. Cheng and Parvin used a breast organoid model and found that matrix stiffness within the high mammographic density range and X-ray radiation had approximately additive effects on aberrant colony formation frequency [45].

These predictive accuracies should be interpreted cautiously. The available evidence derives from relatively small, single- or few-center cohorts, with limited external validation and potential selection bias; reproducibility across laboratories, organoid derivation success rates and assay turnaround times remain incompletely defined. High concordance in a research biobank does not by itself demonstrate clinical usefulness, and prospective multicenter validation, together with standardized protocols and regulatory frameworks, is required before organoid-based predictive assays can be implemented in routine clinical practice.

Tumor organoids represent the direction with the clearest clinical translational value in radiation research. Key breakthroughs include: first, directly linking organoid responses to patient clinical outcomes, with rectal cancer organoid prediction accuracy exceeding 84% and combined treatment prediction accuracy exceeding 92%, transforming organoids from basic research tools into potential companion diagnostic tools; second, revealing that tumor regrowth originates from a small subset of high-Wnt-activity cells, providing a cellular explanation for post-radiation recurrence mechanisms; third, incorporating microenvironmental cells, immune cells, and vascular components into tumor organoids to more closely approximate in vivo conditions; fourth, through AC, IDH1, and other target studies, demonstrating that organoids can be used for radiosensitizer screening and individualized targeted therapy.

## 10. Conclusions

Collectively, organoid applications in radiation research cover three levels: normal tissue radiation injury mechanisms, radioprotection and radiosensitization, and patient individualized response prediction. Core advantages include: derivation from human tissues or iPSCs with the retention of organ-specific cellular composition and three-dimensional structure; amenability to gene editing and lineage tracing; relatively short time from organoid establishment to drug or radiation testing with phenotypic readouts; and retention of genomic and transcriptomic features of primary tumors in PDOs for individualized treatment testing.

In terms of new contributions and key breakthroughs, organoids have advanced radiation research in several ways. First, radiation response research has moved from two-dimensional cell lines to human three-dimensional tissue levels, partially addressing species differences in normal tissue radiosensitivity. Second, microenvironmental signals have been incorporated into radiation regeneration research, demonstrating that immune cells, neurons, mesenchymal cells, and microbial metabolites collectively regulate post-radiation repair. Third, epigenetic reprogramming, stem cell competition, and bystander effects have been introduced into radiation biology, expanding the boundaries of the traditional DNA damage paradigm. Fourth, organoid responses have been directly linked to patient clinical outcomes, positioning organoids as potential predictive biomarkers and companion diagnostic tools. Fifth, radiation quality, dose rate, and FLASH effects have been integrated with three-dimensional tissue biology, providing biological readouts for radiotherapy technology optimization (Figure 1).

The central therapeutic challenge in radiation oncology is to protect normal tissues without shielding the tumor. Organoid studies differ substantially in how far they have addressed this trade-off. Interventions with demonstrated tissue selectivity include BCN057, which rescues normal Lgr5^+^ ISCs after abdominal irradiation and reduces mortality but provides no radioprotection to colon tumor tissue, thereby improving the therapeutic ratio in preclinical models [26]. In contrast, agents such as Me6TREN, SIRT1 inhibitors and the microbial metabolite I3A have shown protective or pro-regenerative effects in normal intestine or intestinal organoids, but their selectivity relative to tumor tissue has not been established [27,28,34]. The Wnt paradox illustrates the problem directly. Pro-regenerative Wnt/β-catenin activation promotes ISC reproliferation and crypt fission in normal epithelium [27,33,34], yet a small subpopulation of high-Wnt-activity cells can initiate tumor regrowth after irradiation [49]. A strategy that globally amplifies Wnt signaling may therefore accelerate repair, and simultaneously, favor tumor recurrence. The most rigorous way to resolve this is paired screening in matched normal-tissue and tumor organoids derived from the same patient, which allows the therapeutic gain of a candidate intervention to be quantified rather than assumed. Such paired normal/tumor organoid biobanks are still scarce, and their establishment should be a priority.

The studies summarized here vary widely in total dose, dose rate, fractionation and assessment time (Table 1). Many use single high-dose exposures, for example, 20 Gy in cardiac organoids [23] or 2 Gy in brain and muscle organoids [12,37], that do not reproduce the fractionated schedules used clinically. Conversely, some studies deliberately examined low-dose and low-dose-rate conditions relevant to radiological protection [6,7]. These differences have direct consequences for translational interpretation: DNA repair kinetics, lineage shifts, senescence and inflammatory responses all depend on dose rate and fractionation, and conclusions drawn from acute single exposures cannot be extrapolated to conventionally fractionated radiotherapy or to HDR conditions. Table 1 therefore distinguishes clinically relevant schedules from experimental exposures, and future studies should report dose, dose rate, fractionation and assessment time in a standardized manner.

Organoid platforms may be particularly valuable in high-dose-rate (HDR) brachytherapy, where steep dose gradients and heterogeneous dose distributions create distinctive radiobiological and dosimetric challenges. Recent work has highlighted methodological gaps in preclinical HDR brachytherapy research, particularly regarding the integration of advanced three-dimensional biological models with accurate dosimetry [53]. Organoids combined with precise dose mapping could help address these gaps by linking local absorbed dose to spatially resolved biological responses, thereby improving the investigation of radiation responses under clinically relevant conditions. However, the current models have several limitations. First, most organoids lack vasculature, immune cells, and innervation, making it difficult to fully recapitulate the in vivo microenvironment. This limitation is not merely technical: it directly constrains which biological questions can be answered. Inflammatory signaling cannot be fully studied without resident macrophages, lymphocytes and cytokine networks; fibrosis cannot be faithfully modeled without fibroblasts, endothelial cells and perfusion; regeneration cannot be fully understood without neural and mesenchymal niche signals; and bystander effects, which depend on intercellular signaling across cell types, are likely underestimated in homogeneous epithelial organoids. Co-culture systems and organ-on-chip platforms that incorporate immune, vascular and neural components are therefore not optional refinements but prerequisites for studying these processes. Second, animal-derived matrices such as Matrigel have undefined composition and immunogenicity issues that limit clinical translation. Third, organoid culture time and cost remain high, and standardization and throughput need improvement. Fourth, the consistency of normal tissue organoid radiation responses with in vivo findings requires further validation, particularly for stem cell competition and bystander effects under low-dose and low-dose-rate conditions. Fifth, clinical evidence for PDO prediction of chemoradiation responses is most robust for rectal cancer, with other tumor types requiring more prospective clinical trials. Sixth, batch-to-batch variation and culture condition differences may affect the reproducibility of radiation response readouts. Finally, experimental marker-γ-H2AX foci, organoid formation efficiency, collagen deposition, and calcium transients do not always reflect actual tissue injury or organ function, and functional validation remains essential.

Possible improvement strategies include: developing composite organoids containing immune, vascular and neural components, or reconstructing the microenvironment through co-culture and microfluidic chips; replacing Matrigel with collagen, synthetic hydrogels, or patient-derived matrices; establishing paired normal tissue and tumor organoid biobanks for assessing therapeutic ratio; combining single-cell multi-omics, spatial transcriptomics, and metabolic imaging to dissect cellular heterogeneity in radiation responses; developing automated, microfluidic, and high-throughput culture platforms to improve reproducibility and standardization; promoting multicenter prospective clinical trials to validate the clinical utility of organoids for predicting chemoradiation responses; and establishing quality control standards and reporting guidelines for organoid radiation response.

Organoids have moved radiation research from two-dimensional cell lines to human three-dimensional tissue and have begun to link mechanistic findings with patient outcomes. However, a clear distinction must be maintained between experimentally demonstrated applications and potential future clinical uses. The most mature and best-supported application is the prediction of chemoradiation response in locally advanced rectal cancer, where concordance exceeds 84% and combined-treatment prediction exceeds 92% [3,4]; even here, validation remains limited and clinical implementation faces unresolved logistical and regulatory barriers. The most mechanistically informative area is intestinal organoid research, where niche crosstalk, epigenetic reprogramming, stem-cell competition and bystander effects have been dissected in detail. In contrast, applications in brain, heart, esophagus, muscle, thyroid and hematopoietic systems remain largely at the proof-of-principle stage.

The main knowledge gaps are: (i) the absence of vascular, immune and neural components; (ii) undefined and immunogenic matrices; (iii) non-standardized and often non-clinically relevant irradiation protocols; (iv) limited donor diversity and external validation; and (v) scarce data on whether candidate radioprotectors preserve tumor control. Priorities for future research should therefore be: composite and vascularized organoids; paired normal/tumor organoid biobanks for therapeutic-ratio assessment; standardized, clinically relevant irradiation schemes including fractionation and HDR conditions; multi-omics together with functional readouts such as electrophysiology and contractility; and multicenter prospective trials with agreed quality-control and reporting standards. With these advances, organoids are expected to become an important platform for radiation biology research and precision radiation oncology, reducing animal experimentation while providing more effective individualized treatment strategies for cancer patients (Figure 2).

## Figures and Tables

**Figure 1:**
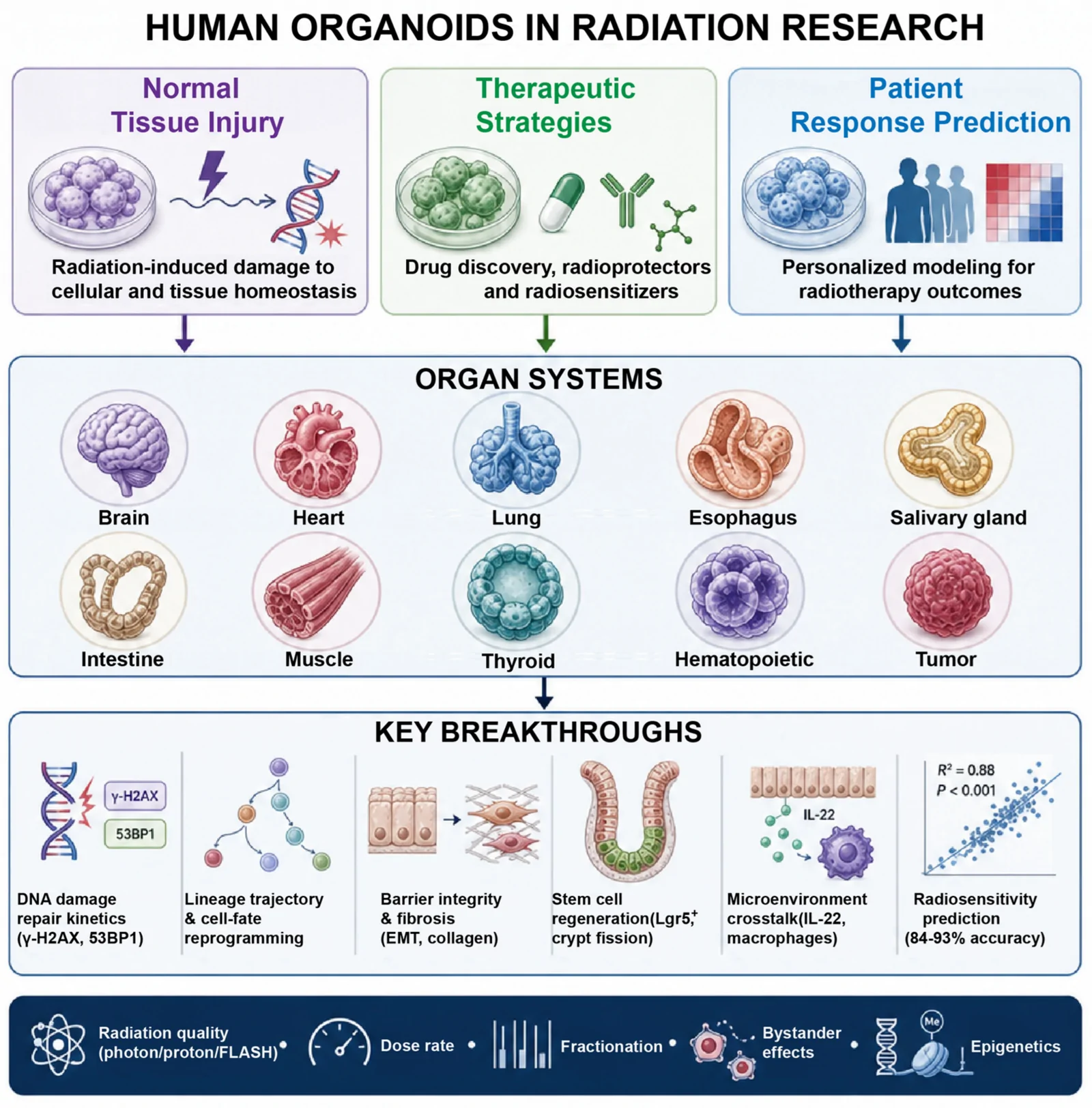
Organoid applications across organ systems in radiation research. The figure emphasizes mechanistic pathways: DNA damage repair kinetics, lineage reprogramming, niche crosstalk, stem-cell competition, bystander signaling and epigenetic remodeling, across the ten organ systems discussed. Portions of the graphical elements were generated using the gpt-image-2.0 model via the AI-Zhitu platform (https://ai.mac89.com/app/text-to-image; accessed on 5 October 2026), and were subsequently substantially edited, assembled, and finalized by the authors in Adobe Illustrator. No AI was used to generate or alter any experimental data. The authors reviewed the final figures and take full responsibility for their content.

**Figure 2:**
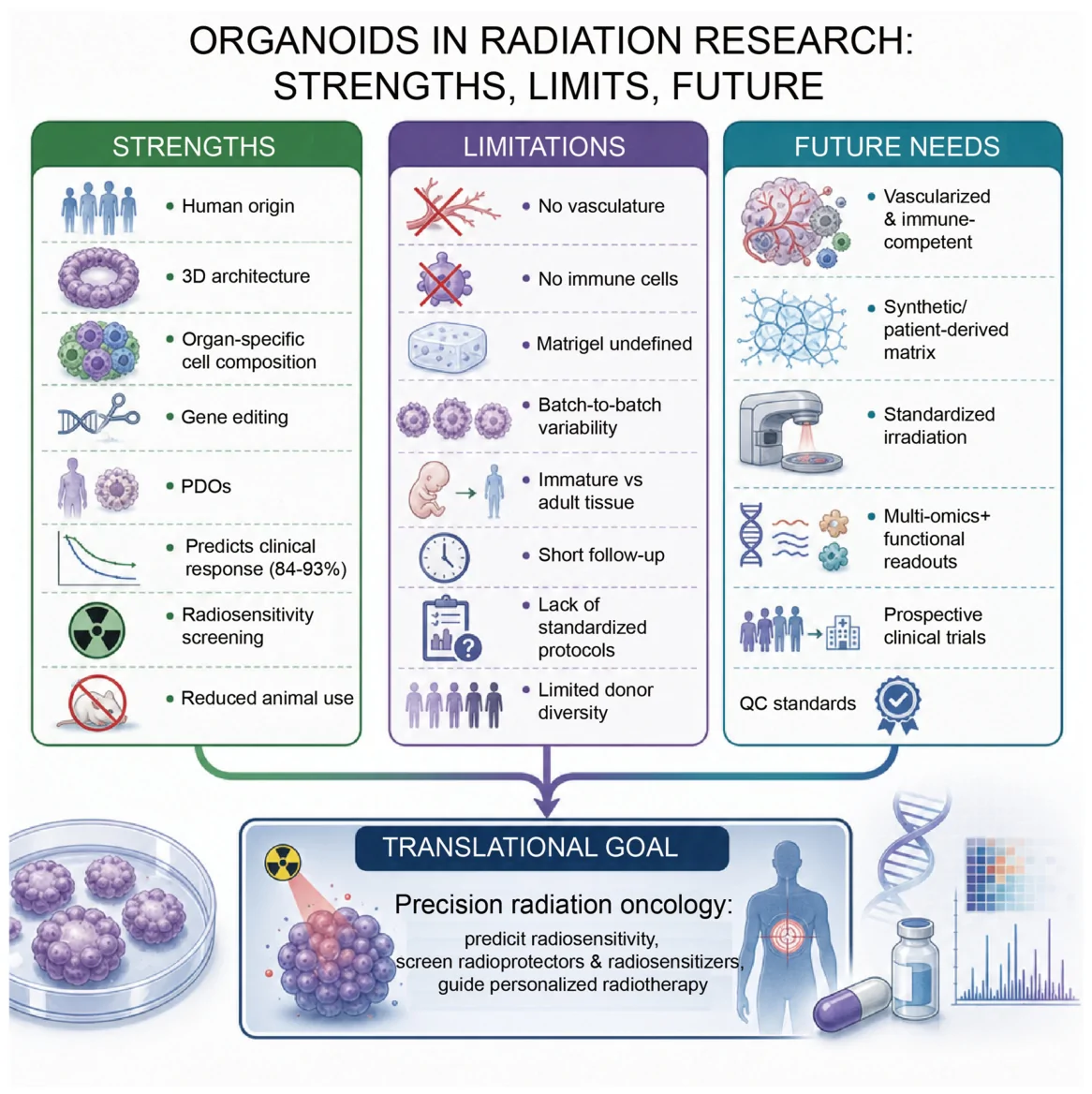
Strengths, limitations and future directions of organoids in radiation research. The figure emphasizes translational challenges: model complexity, matrix standardization, irradiation protocols, validation and clinical implementation, and the corresponding strategies discussed in Section 10. Portions of the graphical elements were generated using the gpt-image-2.0 model via the AI-Zhitu platform (https://ai.mac89.com/app/text-to-image; accessed on 5 October 2026), and were subsequently substantially edited, assembled, and finalized by the authors in Adobe Illustrator. No AI was used to generate or alter any experimental data. The authors reviewed the final figures and take full responsibility for their content.

**Table 1:** Summary of human organoid models in radiation research: cellular origin, irradiation conditions, principal endpoints and key findings.

Organ System	Organoid Model (Origin)	Species	Radiation Type/Dose	Principal Endpoints	Key Findings	Ref.
Brain	Cerebral organoid (hESC)	Human	γ-ray; dose per study	Neuroepithelium integrity, neurite outgrowth, ECAR	Rapamycin/minocycline partially rescue radiation injury	[11]
Brain	Forebrain organoid (hiPSC)	Human	2 Gy, single	γ-H2AX/53BP1 foci kinetics	DSBs mostly resolved by 18 h; faster in progenitors	[12]
Brain	Brain organoid (hiPSC)	Human	Single dose	DNA damage/repair kinetics	Repair kinetics as surrogate for radiosensitivity	[13]
Brain	Brain organoid (hiPSC)	Human	Proton; 0.5 or 2 Gy	Transcriptome, lineage genes	Time/dose-dependent DNA damage; lineage shifts at 48 h	[14]
Brain	Brain organoid (hiPSC)	Human	Single dose	NPC/neuron/astrocyte/OL loss, gliosis	Clonidine reduces cell loss and DNA damage	[15]
Brain	Cerebral organoid (hiPSC)	Human	Single dose	DNA damage, cell death, microcephaly	DMSO/ascorbic acid alleviates damage	[16]
Brain	Cortical organoid (hiPSC)	Human	Single and fractionated	Lineage trajectories, cytokines, layering, synapses	Radiation reshapes lineage; NSPP/amisulpride attenuate	[17]
Brain	Cortical organoid (hiPSC)	Human	Photon vs. plateau proton vs. SOBP	Synaptic signaling genes, network function	SOBP-specific downregulation of development/synaptic genes	[18]
Brain	Rat brain organotypic slice + breast carcinoma	Rat	HIGS-FLASH electrons	Tumor growth, cytokines, microglia	FLASH inhibits tumor growth with differential microglial changes	[19]
Lung	Lung organoid (hiPSC), matrix-free	Human	X-ray; single	Cell-type radiosensitivity, senescence genes	Differential radiosensitivity among lung cell types	[1]
Lung	ALI-differentiated bronchial epithelium	Human	Single	Organoid formation capacity	Reduced for ≥7 days without overt cytotoxicity	[20]
Lung	Lung organoid (hESC)	Human	Repeated irradiation	Proliferation, barrier, EMT, fibrosis markers	RIPF features; pirfenidone reduces TGF-β/α-SMA/COL1A2	[21]
Lung	Lung organoid	Human	0.01 Gy, low dose	Transcriptomics + metabolomics	Glycerophospholipid metabolism affected	[22]
Heart	Cardiac organoid	Human	X-ray; 20 Gy, single (model-specific)	Viability, metabolism, Ca^2+^ transients, mitochondria	UC-MSC-sEVs protect via OXPHOS/p53	[23]
Esophagus	3D esophageal organoid	Murine	High-LET Fe vs. Cs; 0.1 Gy	Squamous differentiation, 53BP1 foci	High-LET more persistent changes; bystander effects	[8]
Salivary gland	Salivary gland organoid	Human (+mouse transplant)	Single	Self-renewal, MUC5B, salivation	Collagen culture preserves phenotype; transplantation restores salivation	[24]
Intestine	Small intestinal organoid	Mouse/human	γ-ray	Damage–repair, regeneration	HNF4α as upstream regulator of regeneration	[25]
Intestine	Intestinal organoid	Mouse/human	Abdominal irradiation	ISC survival, crypt regeneration, mortality	BCN057 protects normal ISCs but not colon tumor	[26]
Intestine	Intestinal organoid	Mouse	Single	Crypt survival, organoid formation	NOD2 deficiency impairs regeneration; Me6TREN promotes via β-catenin	[27]
Intestine	Intestinal organoid	Mouse/human	Single	ISC survival, p53 acetylation	SIRT1 inhibition improves survival (0% → 88.89% in mice)	[28]
Intestine	Intestinal organoid	Mouse/human	Single	ISC reproliferation, crypt–villus restoration	MSC-CM acts via Wnt/Notch	[29]
Intestine	Intestinal organoid	Mouse/human	Single	ISC growth, regeneration	ILC-derived IL-22 acts via STAT3	[30]
Intestine	Intestinal organoid	Mouse	Single	Fetal-like reprogramming, proliferation	Macrophage Nrg1/Spp1 required for regeneration	[31]
Intestine	Intestinal organoid	Mouse/human	Single	Secretory differentiation	VIP acts via p38 MAPK	[32]
Intestine	Intestinal organoid	Mouse	Single	Crypt fission, regularity	WNT4/ROR2 promotes symmetric fission	[33]
Intestine	Intestinal organoid	Mouse/human	Single	Proliferation, probiotic abundance	I3A acts via AhR/IL-10/Wnt	[34]
Intestine	Intestinal organoid	Mouse/human	Single	Pyroptosis, homeostasis	NR inhibits GSDME pyroptosis via SIRT1	[35]
Intestine	Intestinal organoid	Mouse	High/low dose rate; >1 Gy, 0.1 Gy, 1 Gy	OFP, stemness	Dose-rate-dependent changes in stemness	[6,7]
Intestine	Hyper-organoid	Mouse/human	Single	Hyperplastic crypt regeneration	VPA/EPZ6438 + YAP reprogramming	[5]
Intestine	Intestinal organoid	Human	Proton vs. photon	Transcriptome	Distinct molecular responses; fetal TFs, serotonin nodes	[36]
Muscle	hSMO (hPSC)	Human	2 Gy, single	Expansion, differentiation, γ-H2AX/53BP1	PAX7^+^ cells particularly radiosensitive	[37]
Thyroid	Thyroid tissue organoid	Fischer rat	3 Gy γ-ray	Architecture, actin, follicular content, TGF-β	Architectural disruption; active TGF-β release	[38]
Thyroid	Thyroid organoid (hiPSC, PTEN variants)	Human	Single	p53 network activation	PTEN G132D vs. M134R differs in p53 response	[39]
Thyroid	Tissue-derived thyroid organoid	Human	Single	IFN-β signaling, apoptosis	IFN-β mediates inflammation; protects apoptosis but reduces progenitor potential	[40]
Hematopoietic	HSPC + niche cells in GelMA	Human	Single	ROS, γ-H2AX, apoptosis	G-CSF improves HSPC survival and myeloid regeneration	[41]
Tumor	Colorectal cancer organoid	Human	Sublethal/high dose	Proliferation, stemness, regrowth	High-Wnt subset initiates regrowth	[42]
Tumor	Rectal cancer organoid (PDO)	Human	Chemoradiation	Response prediction	84.43% accuracy, 78.01% sensitivity, 91.97% specificity	[3]
Tumor	Rectal cancer organoid (PDO)	Human	Combined treatment	Response prediction	92.50%/93.75% accuracy (discovery/validation)	[4]
Tumor	HNSCC organoid biobank	Human	Radiotherapy	TP53/HPV status, radiosensitivity	TP53 loss alone insufficient; HPV E6/E7 mediates sensitivity	[43]
Tumor	Bladder cancer organoid	Human	RT + PDT	Cytotoxicity, T-cell migration	Synergistic tumor-selective killing	[44]
Tumor	Breast organoid (mammographic density)	Human	X-ray	Aberrant colony formation	Matrix stiffness + radiation additive	[45]

## Data Availability

No new data were created or analyzed in this study. Data sharing is not applicable to this article.

## Abbreviations

The following abbreviations are used in this manuscript:

RT	Radiotherapy
LDR	Low-dose radiation
DNA	Deoxyribonucleic acid
ROS	Reactive oxygen species
iPSCs	Induced pluripotent stem cells
PDO	Patient-derived organoid
γ-H2AX	Gamma-H2A histone family member X
53BP1	Tumor protein p53 binding protein 1
Gy	Gray
DMSO	Dimethyl sulfoxide
NSPP	Not otherwise specified in the text
SOBP	Spread-out Bragg peak
FLASH	FLASH radiotherapy
HIGS-FLASH	High instantaneous dose rate FLASH
LET	Linear energy transfer
MAPK	Mitogen-activated protein kinase
SAPK	Stress-activated protein kinase
hSGOs	Human salivary gland organoids
MUC5B	Mucin 5B
ISCs	Intestinal stem cells
Lgr5	Leucine-rich repeat-containing G-protein coupled receptor 5
HNF4α	Hepatocyte nuclear factor 4 alpha
NOD2	Nucleotide-binding oligomerization domain-containing protein 2
Me6TREN	Tris[2-(dimethylamino)ethyl]amine
SIRT1	Sirtuin 1
MSC-CM	Mesenchymal stem cell-conditioned medium
MSCs	Mesenchymal stem cells
Wnt	Wingless-related integration site
Notch	Notch signaling pathway
ILC	Innate lymphoid cell
IL-22	Interleukin-22
STAT3	Signal transducer and activator of transcription 3
Nrg1	Neuregulin 1
Spp1	Secreted phosphoprotein 1
VIP	Vasoactive intestinal peptide
p38 MAPK	p38 mitogen-activated protein kinase
WNT4	Wingless-related integration site 4
ROR2	Receptor tyrosine kinase-like orphan receptor 2
I3A	Indole-3-aldehyde
AhR	Aryl hydrocarbon receptor
IL-10	Interleukin-10
NR	Nicotinamide riboside
GSDME	Gasdermin E
OFP	Organoid forming potential
VPA	Valproic acid
YAP	Yes-associated protein
TGF-β1	Transforming growth factor beta 1
p-Smad3	Phosphorylated Smad3
hSMOs	Human skeletal muscle organoids
PAX7	Paired box 7
RNA	Ribonucleic acid
CRISPR-Cas9	Clustered regularly interspaced short palindromic repeats-CRISPR-associated protein 9
DTC	Differentiated thyroid cancer
PTEN	Phosphatase and tensin homolog
WT	Wild type
AKT	Protein kinase B
MDM2	Mouse double minute 2 homologs
IFN-β	Interferon beta
PHTS	PTEN hamartoma tumor syndrome
CD34	Cluster of differentiation 34
HSPCs	Hematopoietic stem/progenitor cells
hESCs	Human embryonic stem cells
GelMA	Gelatin methacryloyl
G-CSF	Granulocyte colony-stimulating factor
BM-MSCs	Bone marrow-derived mesenchymal stem cells
TNF-α	Tumor necrosis factor alpha
IFNγ	Interferon gamma
AC	Acid ceramidase
PARP-1	Poly (ADP-ribose) polymerase 1
IDH1	Isocitrate dehydrogenase 1
LARC	Locally advanced rectal cancer
RCO	Rectal cancer organoid
TP53	Tumor protein p53
HPV	Human papillomavirus
E6/E7	HPV E6/E7 oncoproteins
AT1	Alveolar type 1 cells
AT2	Alveolar type 2 cells
EMT	Epithelial–mesenchymal transition
RILI	Radiation-induced lung injury
RIPF	Radiation-induced pulmonary fibrosis
RIHD	Radiation-induced heart disease
sEVs	Small extracellular vesicles
X-ray	X-radiation
γ-rays	Gamma rays
UV	Ultraviolet
3D	Three-dimensional
2D	Two-dimensional
p53	Tumor protein p53
TGF-β	Transforming growth factor beta
α-SMA	Alpha-smooth muscle actin
COL1A2	Collagen type I alpha 2 chain
